# Estimating prognosis and palliation based on tumour marker CA 19-9 and quality of life indicators in patients with advanced pancreatic cancer receiving chemotherapy

**DOI:** 10.1038/sj.bjc.6605929

**Published:** 2010-09-28

**Authors:** J Bernhard, D Dietrich, B Glimelius, V Hess, G Bodoky, W Scheithauer, R Herrmann

**Affiliations:** 1 SAKK Coordinating Center, Bern, Switzerland; 2 Inselspital, Bern University Hospital, Bern, Switzerland; 3 University of Uppsala, Uppsala, Sweden; 4 University Hospital, Basel, Switzerland; 5 Szt. László Hospital, Budapest, Hungary; 6 Medical University of Vienna, Vienna, Austria

**Keywords:** CA 19-9 tumour marker, quality of life, prognostic factor, palliation, advanced pancreatic carcinoma

## Abstract

**Background:**

To investigate the prognostic value of quality of life (QOL) relative to tumour marker carbohydrate antigen (CA) 19-9, and the role of CA 19-9 in estimating palliation in patients with advanced pancreatic cancer receiving chemotherapy.

**Methods:**

CA 19-9 serum concentration was measured at baseline and every 3 weeks in a phase III trial (SAKK 44/00–CECOG/PAN.1.3.001). Patients scored QOL indicators at baseline, and before each administration of chemotherapy (weekly or bi-weekly) for 24 weeks or until progression. Prognostic factors were investigated by Cox models, QOL during chemotherapy by mixed-effect models.

**Results:**

Patient-rated pain (*P*<0.02) and tiredness (*P*<0.03) were independent predictors for survival, although less prognostic than CA 19-9 (*P*<0.001). Baseline CA 19-9 did not predict QOL during chemotherapy, except for a marginal effect on pain (*P*<0.05). Mean changes in physical domains across the whole observation period were marginally correlated with the maximum CA 19-9 decrease. Patients in a better health status reported the most improvement in QOL within 20 days before maximum CA 19-9 decrease. They indicated substantially less pain and better physical well-being, already, early on during chemotherapy with a maximum CA 19-9 decrease of ⩾50% *vs* <50%.

**Conclusion:**

In advanced pancreatic cancer, pain and tiredness are independent prognostic factors for survival, although less prognostic than CA 19-9. Quality of life improves before best CA 19-9 response but the maximum CA 19-9 decrease has no impact on subsequent QOL. To estimate palliation by chemotherapy, patient's perception needs to be taken into account.

Given the poor prognosis of advanced pancreatic cancer and the symptom burden, symptom palliation and balancing the trade-offs between quality of life (QOL) and survival are of paramount importance in these patients. This is particularly the case when discussing palliative chemotherapy. Quality of life as perceived by the patient is not only an important treatment outcome but also a prognostic factor (Gotay *et al*, 2008; Montazeri, 2009; Quinten *et al*, 2009).

Prognostic factors are especially valuable in this situation. Pre-treatment concentration of the tumour marker carbohydrate antigen (CA) 19-9 is a strong independent prognostic factor for survival in advanced pancreatic cancer (Micke *et al*, 2003; Maisey *et al*, 2005; Hess *et al*, 2008). Quality of life also predicts survival in these patients (Gupta *et al*, 2006; Robinson *et al*, 2008). The contribution of QOL relative to CA 19-9, that is, its clinical significance as an independent prognostic factor, is not known.

An early decrease in CA 19-9 concentration during chemotherapy (i.e., a CA 19-9 response) might not only serve as prognostic marker but also as an early marker of tumour response. This would discriminate patients likely to benefit from continued treatment from those who would not. However, in our recent phase III trial (Herrmann *et al*, 2007) a decrease in CA 19-9 concentration during chemotherapy was not significantly associated with lengthened survival compared with those patients who did not have a corresponding decrease (Hess *et al*, 2008).

Whether a decrease in CA 19-9 concentration is associated with palliation as perceived by the patient is not known. Given that palliation and thus QOL is a primary treatment goal in these patients, the question arises whether the CA19-9 response, at any time for an individual patient, may serve as decision aid for continuing, switching, or stopping chemotherapy.

We investigated the prognostic value of QOL relative to CA 19-9, and the role of CA 19-9 in estimating palliation in patients with advanced pancreatic cancer receiving chemotherapy within a randomised controlled clinical trial (Herrmann *et al*, 2007; Bernhard *et al*, 2008).

## Patients and methods

### The trial

All patients, with histologically proven, locally advanced, or metastatic adenocarcinoma of the pancreas, treated in the international phase III trial SAKK 44/00–CECOG/PAN.1.3.001 (Herrmann *et al*, 2007) with an elevated baseline tumour marker CA 19-9 were included in this study. Patients had a Karnofsky Performance Status (KPS) >60, were naive to chemotherapy for advanced disease, and had not received any adjuvant radio- or radiochemotherapy 12 months before inclusion. Patients were stratified by KPS (90–100 *vs* 60–80), disease extent (locally advanced v metastatic), presence or absence of pain, and enrolling centre and then randomly assigned to GemCap (oral capecitabine 650 mg m^−2^ twice daily on days 1–14 plus gemcitabine 1000 mg m^−2^ 30-min infusion on days 1 and 8 every 3 weeks) *vs* Gem (gemcitabine 1000 mg m^−2^ 30-min infusion weekly for 7 weeks, followed by a 1-week break, and then weekly for 3 weeks every 4 weeks). Treatment was continued until disease progression or for a maximum of 24 weeks, except in the case of unacceptable toxicity. Treatment could be resumed later at the discretion of the investigator. Treatment decisions were based on clinical and radiographic grounds, not on CA 19-9 values. Informed consent was obtained from all patients and ethical committee approval was given by all participating centres. Trial protocol and conduct are described elsewhere (Herrmann *et al*, 2007).

### CA 19-9 and tumour response assessment

Carbohydrate antigen 19-9 measurements were performed at baseline (within 3 days of treatment start) and every 3 weeks thereafter. The upper limit of laboratory normal (ULN) range was 18–37 U ml^−1^, depending on the methods used by the different laboratories involved (La’ulu and Roberts, 2007). Baseline and follow-up measurements for any given patient were carried out at the same laboratory and by use of the same testing method. Patients with a CA 19-9 value >1.0 × ULN were included for baseline analyses (Hess *et al*, 2008). For the assessment of CA 19-9 response, only patients with baseline values >1.5 × ULN and at least one follow-up value on or after day 42 were included (Hess *et al*, 2008). The CA 19-9 best response was defined as the lowest concentration measured at any time for each individual patient compared with the baseline value. A CT scan was performed at baseline, every 6 weeks during chemotherapy and every 9 weeks during follow-up. Tumour response (i.e., best response during treatment or follow-up) was assessed according to the Response Evaluation Criteria In Solid Tumors (Therasse *et al*, 2000).

### QOL assessment

Quality of life was a secondary end point (Bernhard *et al*, 2008). Patients were asked to complete a QOL form at baseline and weekly for the first 7 weeks, and subsequently before each administration of Gem for 24 weeks from random assignment. The forms were completed at the hospital and, with the exception of the baseline, before diagnostic procedures.

For this analysis, we used the following global linear-analogue self-assessment (LASA) indicators sensitive to the wide spectrum of reactions seen in patients on chemotherapy: Physical well-being (Butow *et al*, 1991), mood (Butow *et al*, 1991; Hurny *et al*, 1996), coping effort (Butow *et al*, 1991; Hurny *et al*, 1993), and functional performance (Bernhard *et al*, 1999). The indicators for physical well-being, mood, coping effort, and functional performance were sensitive to tumour response in metastatic colorectal cancer (Borner *et al*, 2005). The issue of high psychological distress in pancreatic cancer (Zabora *et al*, 2001) was covered by the mood and coping indicators, which are sensitive to mood disorders and psychosocial dysfunction (Bernhard *et al*, 2001). Specific LASA indicators were used for pain (Fishman *et al*, 1987) and tiredness (Bernhard *et al*, 1999). The restriction to a few key indicators instead of a standard assessment was based on feasibility considerations for the intensive longitudinal assessment schedule (Bernhard *et al*, 2008).

### Statistical analysis

Quality of life forms filled in >3 days before or after day 1 of a cycle were excluded. We report means of untransformed data (scale range: 0–100; higher scores: better condition). A mean change of ⩾8 points from baseline was defined as clinically meaningful (Sloan and Dueck, 2004).

Associations among the stratification factors, baseline CA 19-9 and QOL scores (grouped by medians), and tumour response (Therasse *et al*, 2000) were investigated by *χ*^2^-tests, associations among the continuous QOL scores by Spearman correlations. We used *R*⩾0.7 as criterion for multi-collinearity (Van Steen *et al*, 2002).

A Cox model for overall survival was calculated with stratification factors, treatment arm, and baseline CA 19-9 as predictors. Then the QOL indicators were added individually to the model, first with continuous and second with grouped scores (cut-off points: 33 and 67% quantile of total sample, intermediate group as reference). The findings of the grouped scores were more consistent due to nonlinear effects, and are presented here. A second Cox model was calculated for all QOL indicators without clinical factors and a third model with clinical factors and QOL indicators.

Associations between QOL changes and maximum CA 19-9 decrease (both to baseline) were explored by Spearman correlations. The prognostic value of CA 19-9 baseline concentration and CA 19-9 maximal decrease for QOL during chemotherapy was investigated by a linear mixed-effects model for each indicator taking into account time effects on QOL, split by monthly treatment duration (Bernhard *et al*, 2008).

The time before and after best CA 19-9 response was divided into four periods. They were defined as the smallest intervals coinciding with weekly QOL assessments and yielding robust associations. Mean QOL changes by maximum CA 19-9 decrease of <50 *vs* ⩾50% were investigated by a linear mixed-effects model over these periods for each indicator. Baseline QOL was used as a covariate and the number of forms included in a patient's period mean as a weight variable.

## Results

### Sample description and patient characteristics

Out of 319 randomised patients, four did not receive trial treatment and four had no visit forms. Of the remaining 311 patients, 96% had a baseline QOL form. Of all expected QOL forms under treatment, we received 86% (*N*=3033/3536). Of those, 95% were filled in at day 1 or within 3 days before a chemotherapy cycle. Participants and non-participants at the last scheduled QOL assessment (week 23) were similar regarding age, sex, disease status, KPS, and pain requiring medication at random assignment (data not shown). A majority of patients had metastatic disease and pain requiring analgesic medication (Table 1).

According to our criteria for CA 19-9 evaluation, 247 patients were assessable at baseline. There were no apparent differences between these patients and the total sample (data not shown), and between those with normal and increased (median=59 × ULN) CA 19-9 baseline concentration (Table 1). Of the 247 patients with increased concentration, 175 had at least one follow-up assessment of CA 19-9 on or after day 42, and were assessable for best CA 19-9 response.

### Associations among patient characteristics, CA 19-9, and QOL at baseline

The only tendency for an association between the baseline characteristics and normal *vs* increased CA 19-9 concentration was the proportion of patients requiring analgesic medication according to the treating physician (Table 1), with a higher proportion (70 *vs* 58%) in the group with increased concentration (*P*=0.12), but no difference in average analgesic consumption at this time.

The baseline QOL scores are shown by CA 19-9 concentration in Table 2. Those for coping effort, tiredness, and mood were particularly impaired. There was no substantial association between QOL and CA 19-9 or extent of disease at baseline. Patients with lower KPS had significantly worse scores in all QOL indicators (data not shown). The correlations among the QOL indicators ranged between *R*=0.32 and 0.64. There was no indication for harmful multi-collinearity.

### Prognostic value of baseline QOL

Baseline QOL was not associated with tumour response (CR/PR: *N*=27; SD: *N*=173; PD: *N*=53). Similarly, there was no association between baseline CA 19-9 and tumour response, as reported previously (Hess *et al*, 2008). There was no substantial correlation between baseline QOL and best CA 19-9 response (i.e., absolute value) or maximum (i.e., relative) decrease of CA 19-9.

In the total sample (*N*=319), the median time to progression (TTP) was 4.3 and 3.9 months in patients randomly assigned to GemCap or Gem, respectively (Herrmann *et al*, 2007); that is, the majority of patients failed during our observation period of 6 months. In a Cox model with stratification factors, treatment arm and baseline CA 19-9 >1 × ULN (*N*=247; TTP GemCap and Gem: 4.4 and 3.9 months), the strongest effect was present for CA 19-9, followed by physician-rated KPS and pain requiring analgesic medication, as shown in Table 3. After adding the QOL indicators separately, tiredness (good *vs* intermediate) was additionally prognostic, with less tiredness resulting in a better survival (HR: 0.68, 95% CI: 0.48-0.96, *P*<0.05). Similarly, patients who reported less pain (good *vs* intermediate) had a better survival (HR: 0.69, 95% CI: 0.48–0.98, *P*<0.05). It is important to note that these effects were not linear but discriminated patients with low symptom burden from the others. The remaining indicators were not associated with survival. Pain and tiredness (borderline effect) were also the only prognostic indicators in the model without clinical factors (Table 3).

**Figure 1:**
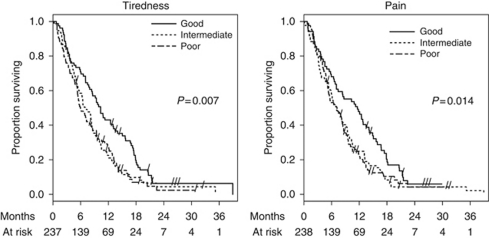
Overall survival by baseline scores of tiredness (cut-off points: 50, 77) and pain (cut-off points: 71, 92). Both indicators have a range from 0 to 100, with higher scores indicating a better condition. *P*-values are based on log-rank test.

Finally, a Cox model was calculated including both the clinical factors and QOL indicators (Table 3). Patient-rated pain intensity was more prognostic than pain requiring analgesic medication according to the treating physician. In contrast, patient-rated functional performance remained unprognostic after exclusion of KPS. Survival according to tiredness and pain intensity is shown in Figure 1. A univariate analysis of all available patients (including CA 19-9 ⩽1 × ULN) showed similar findings.

### Associations between CA 19-9 and QOL during chemotherapy

Mean changes in physical QOL domains across the whole observation period were marginally correlated with the maximum CA 19-9 decrease, ranging from *R*=0.15 (*P*<0.05) for functional performance to *R*=0.28 (*P*<0.001) for physical well-being. To investigate the role of CA 19-9 in estimating palliation, we first explored the prognostic value of CA 19-9 baseline concentration and of maximal decrease for QOL during chemotherapy by mixed-effects models using all available data until failure (*N*=231). The only, but not relevant, difference by baseline CA 19-9 was present in pain: Patients with baseline CA 19-9 below the median indicated marginally less pain on treatment (average score: 8.5 *vs* 8.3 *P*<0.05). The maximum CA 19-9 decrease had no impact on any of the QOL indicators after best response; that is, the scores were similar to those before best response. There was no significant interaction between CA 19-9 baseline scores and maximum decrease (*P*=0.1), time effects on QOL, or time on study treatment.

**Figure 2:**
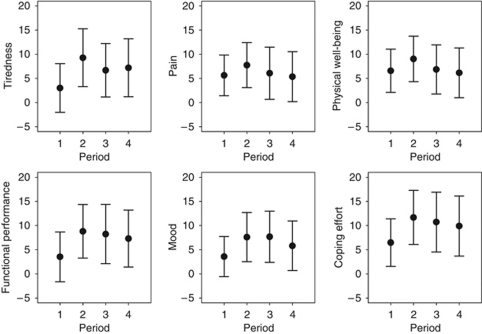
Mean changes (*N*=85) in QOL (95% CI) between baseline and different periods relative to the time point of best CA 19-9 response. Four periods were defined: ⩾21 days (period 1) or <21 days (2) before best response, and <21 days (3) or ⩾21 days (4) after best response. Higher scores indicate an improvement. A meaningful change is defined as a mean change of ⩾8 points from baseline in either direction.

Second, we explored the changes in QOL from baseline according to their timing relative to the best CA 19-9 response in a subsample. Four periods were defined: ⩾21 days (period 1) or <21 days (2) before best CA 19-9 response, and <21 days (3) or ⩾21 days (4) after best response. Eighty-five patients with baseline CA 19-9 ⩾1.5 × ULN, CA 19-9 measurements after day 40, and QOL measurements in all four periods were available (TTP GemCap and Gem: 7.1 and 5.8 months). For a conservative estimate of QOL, all measurements were averaged within each period for each patient and indicator. In this relatively healthy subsample, there was an improvement in all periods and indicators (Figure 2). The largest improvement was within 20 days before the best response of CA 19-9, with mean changes varying between 7.6 for mood and 11.7 for coping effort. After the best CA 19-9 response, QOL tended to decline but remained above baseline.

To estimate the clinical meaning of these changes, each patient's change of each indicator was grouped for each period as ‘better’, ‘stable’, or ‘worse’ relative to baseline, according to our pre-defined criterion (mean change of ⩾8 points from baseline in either direction). With some variation among the periods and indicators, more than two-thirds of the patients reported stable or improving scores regardless of the period. In other words, there was no indication that a minimally important change in QOL was associated with the timing relative to the best CA 19-9 response. It is noteworthy that all these patients received trial chemotherapy in all four periods.

**Figure 3:**
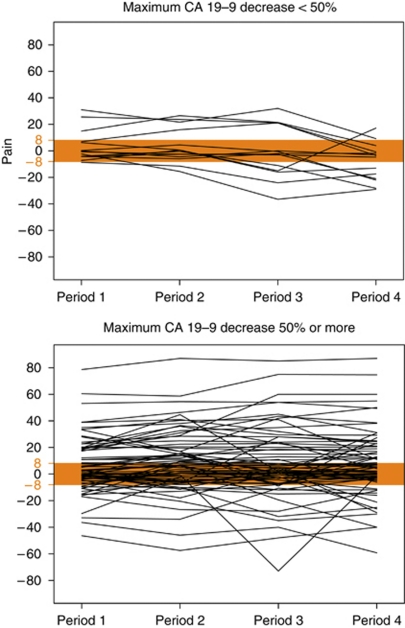
Individual changes in pain between baseline and different periods relative to the time point of best CA 19-9 response, split by the cut-off of <50% (*N*=14) *vs* ⩾50%: (*N*=71) maximum decrease. Four periods were defined: ⩾21 days (period 1) or <21 days (2) before best response, and <21 days (3) or ⩾21 days (4) after best response. Higher scores indicate an improvement. A meaningful change is defined as a mean change of ⩾8 points from baseline in either direction.

Finally, we explored the changes in QOL according to the maximum CA 19-9 decrease in this subsample. Figure 3 shows individual changes in pain across the four periods split by the cut-off of <50 *vs* ⩾50% maximum decrease. Patients with a maximum CA 19-9 decrease of <50% (*N*=14) indicated stable scores or an improvement at beginning, and a declining tendency over time (mean change to baseline=−1). Those with a decrease of at least 50% (*N*=71) showed in average an early improvement in pain, which remained roughly stable (mean change to baseline=10; group difference: *P*<0.005). Similarly, patients with a maximum decrease of at least 50% indicated better physical well-being across all periods, with an overall change in physical well-being of 9 as compared with 0 (*P*<0.05).

Within these four periods, changes in pain and physical well-being were weakly correlated with the maximum CA 19-9 decrease, more prominent after best CA 19-9 response as compared to before (e.g., pain <21 days before: *R*=0.27, *P*<0.05; <21 days after: *R*=0.34, *P*<0.005). To estimate whether this correlation was depending on the initial tumour load, patients were grouped according to the median CA 19-9 concentration at baseline. Only patients with a value below the median (27 × ULN) showed a significant correlation between maximum CA 19-9 decrease and changes in pain within 20 days before (*R*=0.37, *P*<0.05). These patients reported better pain scores at baseline (median: 89.5 *vs* 67.0; *P*<0.05).

In summary, baseline CA 19-9 had no relevant prognostic impact on QOL until treatment failure. There was an association between the maximum CA 19-9 decrease and pain and physical well-being, respectively, but the maximum decrease was not prognostic for these domains: These group differences in pain and physical well-being were present already before the maximum decrease.

## Discussion

We investigated the prognostic value of QOL relative to CA 19-9, and the role of CA 19-9 in estimating palliation in patients with advanced pancreatic cancer receiving chemotherapy within a randomised controlled clinical trial (Herrmann *et al*, 2007; Bernhard *et al*, 2008).

At baseline, less pain and tiredness, that is, less symptom burden, predicted better survival, as shown for various QOL domains in other cancer sites (Gotay *et al*, 2008; Montazeri, 2009; Quinten *et al*, 2009). In advanced disease, mainly physical functioning and symptoms have been prognostic. Pre-treatment fatigue was a dominant prognostic factor in patients with advanced head and neck carcinoma treated with radiotherapy (Fang *et al*, 2004). Similarly, pain and dysphagia were strong prognostic factors in patients with non-small-cell lung cancer (Efficace *et al*, 2006). We did not assess further patient-rated symptoms in our trial. Pain and tiredness showed consistent findings in all models. Their nonlinear association with survival may be confounded by individual analgesic treatment before the baseline assessment. These symptoms may serve as baseline characteristics for patients with advanced pancreatic cancer treated in clinical trials. Their relative contribution is, however, smaller than the effect of pre-treatment concentration of CA 19-9 on survival.

In contrast, baseline CA 19-9 did not predict QOL or time on study treatment, besides a marginal effect on pain. Neither CA 19-9 nor QOL predicted tumour response to chemotherapy. Survival is influenced by different factors than response to chemotherapy, although response impacts on survival. Thus, CA 19-9 and QOL at baseline provide limited information for estimating palliation by chemotherapy.

In patients with a better health status, QOL during chemotherapy differed by the timing relative to the best CA 19-9 response. Twenty days before the best CA 19-9 response, patients reported the most improvement from baseline. The subsequent QOL scores, however, were not affected by the maximum CA 19-9 decrease. The maximum decrease had an effect on physical domains across the whole observation period: Those patients with a better CA 19-9 response had better scores already early on chemotherapy. There was no indication that a minimal important change in QOL was associated with the timing relative to the best CA 19-9 response. Thus, the shift in QOL preceding the best CA 19-9 response and the maximum decrease of CA 19-9 provide little information on the net palliation by chemotherapy. The decision of whether to continue switch or stop chemotherapy cannot be made on the basis of early CA19-9 kinetics alone. The patient's experience of disease and treatment as a whole needs to be taken into account.

In our trial, QOL improved under chemotherapy and deteriorated again before treatment failure was documented by the clinician (Bernhard *et al*, 2008). The majority of those patients receiving only few cycles indicated a benefit from chemotherapy (Bernhard *et al*, 2008). There were no effects by the randomly assigned treatment groups (gemcitabine *vs* gemcitabine plus capecitabine) on survival, QOL, or clinical benefit (Bernhard *et al*, 2008). It remains unclear, whether the palliation as indicated by patients’ QOL was caused by a very brief antitumour effect by chemotherapy or by the conditions of the situation itself, for example, receiving antitumour treatment, supportive care or more steroids than before. Some associations between maximum CA 19-9 decrease and pain or physical well-being may reflect both the impact of anti-tumour or analgesic treatment.

Several limitations of this study have to be noted. The predictive and prognostic value of baseline QOL was specified in the protocol. However, our findings are based on a secondary analysis of a phase III trial. Their clinical validity is restricted by potential selection criteria bias. An observational study would provide more evidence of the prognostic value of baseline QOL. A comprehensive standard QOL assessment would have given supplemental information on symptoms and broader domains of functioning and well-being, but was not feasible for our intensive assessment schedule (Bernhard *et al*, 2008). We hypothesised that the different time schedules used in previous trials in this population contributed to the inconsistent findings on the impact of single-agent gemcitabine on QOL (Bernhard *et al*, 2008). To minimise the potential bias associated with early withdrawal from study treatment and to investigate patients’ underlying trajectories of palliation, we have chosen a weekly or fortnightly assessment for QOL over 6 months that revealed consistent time effects (Bernhard *et al*, 2008). We selected simple indicators as an alternative to a comprehensive questionnaire, which is better suited for widely spaced estimates. There was no indication for multi-collinearity using these indicators (Van Steen *et al*, 2002). Finally, this investigation is based on different subsamples, including patients in a relatively good health status. Overall, however, the findings of the different analyses are consistent.

In conclusion, in advanced pancreatic cancer, pain and tiredness are independent prognostic factors for survival, although they are less prognostic than CA 19-9. Neither CA 19-9 nor QOL did predict tumour response. Baseline CA 19-9 does not predict QOL under chemotherapy. QOL improves before best CA 19-9 response but the maximum decrease has no impact on subsequent QOL. Best CA 19-9 response alone does not provide sufficient information about palliation by chemotherapy. Patient's perception needs to be taken into account.

## Figures and Tables

**Table 1:** Characteristics and quality of life scores of the total sample by tumour marker concentration at baseline

	**Increased tumour marker concentration (*N*=247)** a	**Normal tumour marker concentration (*N*=48)** b
Median age/range (years)	63/26–83	61/36–73
Male/female (%)	47/53	46/54
Extent of disease: locally advanced/metastatic (%)	22/78	17/83
		
*Karnofsky performance status*		
90–100/60–80 (%)	54/46	50/50
Pain requiring medication (%)^c^	70	58
Average baseline analgesic consumption (morphine-equivalent mg) over 4 days (median/range)	0/0–104	0/0–101

Abbreviations: CA=carbohydrate antigen; ULN=upper limit of laboratory normal.

a Increased CA 19-9: >1 × ULN; median (range): 59 (1–90 032).

b Normal CA 19-9: ⩽1 × ULN.

c According to the treating physician.

**Table 2:** Quality of life scores of the total sample by tumour marker concentration at baseline^a^

	**Increased tumour marker concentration (*N*=236–238)** b		**Normal tumour marker concentration (*N*=45–46)** c	
	**Median**	**Range**	**Median**	**Range**
Pain intensity	83	6–100	88	5–100
Tiredness	62	2–100	70	2–100
Physical well-being	71	1–100	72	4–100
Functional performance	70	1–100	62	1–100
Mood	63	0–100	59	3–100
Coping effort	57	0–100	60	4–100

Abbreviations: CA=carbohydrate antigen; ULN=upper limit of laboratory normal.

a Higher scores indicate a better condition (e.g., less coping effort).

b Increased CA 19-9: > 1 × ULN; median (range): 59 (1–90 032).

c Normal CA 19-9: ⩽1 × ULN.

**Table 3:** Prognostic value of baseline factors for overall survival in patients with an increased tumour marker concentration

	**Cox model for clinical factors**			**Cox model for QOL factors**			**Cox model for clinical and QOL factors**		
**Factor**	**Hazard ratio**	**95% CI**	***P***	**Hazard Ratio**	**95% CI**	***P***	**Hazard ratio**	**95% CI**	***P***
*Stratification factor*									
Pain requiring medication	0.73	0.54–0.98	**0.035**				—a		
Locally advanced *vs* metastatic disease	0.81	0.58–1.13	0.222				0.79	0.55–1.11	0.174
Karnofsky performance status of 90–100 *vs* 60–80	0.73	0.56–0.96	**0.025**				0.67	0.48–0.94	**0.02**
Treatment arm GemCap *vs* Gem	0.91	0.69–1.18	0.467				0.83	0.62–1.10	0.184
Tumour marker CA 19-9 low (⩽59 × ULN) *vs* high (>59 × ULN)	0.48	0.36–0.64	**<0.001**				0.41	0.30–0.55	**<0.001**
									
*QOL indicators:*									
*Tiredness*									
Poor *vs* intermediate				1.07	0.74–1.54	0.737	1.04	0.72–1.49	0.847
Good *vs* intermediate				0.7	0.48–1.03	**0.07**	0.65	0.45–0.96	**0.03**
									
*Pain*									
Poor *vs* intermediate				0.87	0.61–1.23	0.429	0.75	0.52–1.09	0.129
Good *vs* intermediate				0.65	0.45–0.93	**0.019**	0.63	0.44–0.92	**0.015**
									
*Physical well-being*									
Poor *vs* intermediate				1.17	0.77–1.77	0.453	1.09	0.72–1.65	0.691
Good *vs* intermediate				1.16	0.79–1.70	0.452	1.33	0.90–1.96	0.157
									
*Functional performance*									
Poor *vs* intermediate				0.8	0.55–1.17	0.246	—b		
Good *vs* intermediate				0.9	0.60–1.35	0.621			
									
*Mood*									
Poor *vs* intermediate				1.01	0.68–1.50	0.946	1.07	0.72–1.59	0.741
Good *vs* intermediate				0.92	0.63–1.33	0.654	0.94	0.64–1.38	0.759
									
*Coping effort*									
Poor *vs* intermediate				0.89	0.61–1.29	0.523	0.76	0.51–1.12	0.161
Good *vs* intermediate				0.96	0.67–1.38	0.836	1.01	0.71–1.43	0.963

Abbreviations: CA=carbohydrate antigen; CI=confidence interval; QOL=quality of life; ULN=upper limit of laboratory normal.

a Pain requiring medication according the treating physician was replaced by patient-rated pain intensity.

b Patient-rated functional performance was replaced by Karnofsky performance status. Bold values indicate statistical significance or borderline effect.
